# Transcriptional and biochemical profiling of defense enzymes in *Citrus sinensis* during salicylic acid and cinnamon mediated suppression of green and blue mold

**DOI:** 10.3389/fpls.2022.1048433

**Published:** 2022-11-03

**Authors:** Anam Moosa, Faisal Zulfiqar, Kadambot H. M. Siddique

**Affiliations:** ^1^ Department of Plant Pathology, Faculty of Agriculture and Environment, The Islamia University of Bahawalpur, Bahawalpur, Pakistan; ^2^ Department of Horticultural Sciences, Faculty of Agriculture and Environment, The Islamia University of Bahawalpur, Bahawalpur, Pakistan; ^3^ The UWA Institute of Agriculture, The University of Western Australia, Perth, WA, Australia

**Keywords:** inhibition, sweet oranges, enzyme, peroxidase, phenyl-alanine ammonia-lyase, polyphenol oxidase, biostimulants

## Abstract

Green and blue mold of citrus are threatening diseases that continuously inflict economic post-harvest loss. The suppressive effect of salicylic (SA) and *Cinnamomum verum* (CV) on green and blue mold of sweet oranges was investigated in this study. Among five tested plant extracts methanolic extract of Cinnamon caused the highest colony growth inhibition of *P. digitatum* and *P. italicum* in an *in vitro* antifungal assay. The methanolic extract of Cinnamon in combination with SA showed the lowest disease incidence and severity of green and blue mold on citrus fruit without affecting the fruit quality. Transcriptional profiling of defense enzymes revealed that the polyphenol oxidase (*PPO*), phenylalanine ammonia-lyase (*PAL*), and peroxidase (*POD*) genes were upregulated in fruit treated with CV, SA, and their combination compared to the control. The treatment SA+CV caused the highest upsurge in *PPO*, *POD*, and *PAL* gene expression than the control. Furthermore, the biochemical quantification of PPO, POD and PAL also revealed a similar pattern of activity. The present findings unravel the fact that the escalation in the activity of tested defense enzymes is possibly associated with the reduced incidence of blue and green molds. In conclusion, the study unveils the promising suppressive potential of SA+CV against green and blue mold by regulating the expression of *PPO*, *POD*, and *PAL* genes. Therefore, these treatments can find a role as safer alternatives to chemicals in the management of post-harvest green and blue mold.

## 1 Introduction

Citrus is the most widely grown fruit crop in Pakistan and constitutes 30% of the total fruit production. It is cultivated throughout the country, but the leading Citrus growing region is Punjab, Pakistan (Siddique and Garnevska, 2018). Pakistan exports a huge amount of Citrus produce, therefore the production loss due to pre-and post-harvest threats remains the biggest challenge affecting the total export of the country. During processing practices, post-harvest diseases cause a huge loss of production. Among them blue and green mold incited by *P. italicum* Wehmer, and *Penicillium digitatum* (Pers.:Fr.) Sacc. are the major post-harvest rots which cause quick deterioration of citrus fruit (Palou et al., 2001; Panebianco et al., 2015). They are ubiquitous pathogens which cause huge economic loss during transportation, processing, and storage and ultimately reduce the storage life of Citrus fruit. The injuries inflicted on the fruit during transportation and storage and the enormous number of airborne conidia aggravate the incidence of Penicillium rot (Frisvad and Samson, 2004; Panebianco et al., 2015).

Currently, the most common practice to control *Penicillium* decay is by using the fungicides. However, the unthrifty use of fungicides and the continuous development of resistance in the pathogen against fungicides is a growing concern for human and environment health (Holmes and Eckert, 1999; Moscoso-Ramírez et al., 2013). The increasing concerns of consumers over health and environment safety and chemical residues in the fruit have prompted the need to look for safer alternatives for fungicides to prevent the loss caused by post-harvest pathogens (Moscoso-Ramírez et al., 2013). Several alternative strategies have been employed to reduce the incidence of post-harvest spoilage of citrus fruit such as physical methods, plant extracts, biological control, radiations, and low-toxicity chemicals (Palou et al., 2001; Palou, 2009; Askarne et al., 2013).

Plants by default possess a natural defense mechanism to cope with the unfavorable biotic and abiotic stresses (Hasanuzzaman et al., 2020; Zulfiqar et al., 2020; Zulfiqar and Ashraf, 2021a; b) which can be induced or enhanced by physical, chemical, and biological elicitors (Ahmed et al., 2021a; Ahmed et al., 2021b; Zulfiqar, 2021; Zulfiqar et al., 2021; Jiang et al., 2021; Zulfiqar et al., 2022). The chemical resistance inducers salicylic acid (SA), benzoic acid (BA), methyl jasmonate (MJ), and jasmonic acid (JA), can alter the way host and pathogens interact with each other. Among resistance inducers, salicylic acid (SA) is an endogenous, natural resistance inducer which regulates defense responses, growth of the plants, and protects from pre-and post-harvest pathogens (Shaat and Galal, 2004; Cao et al., 2006; Iqbal et al., 2012; Giménez et al., 2014; Moosa et al., 2021). Several reports have stated that the application of SA at low concentrations improved the resistance against post-harvest spoilage of various fruit such as sweet cherries (Xu and Tian, 2008), peaches (Xu et al., 2008), mangoes (Zeng et al., 2006), and citrus (Iqbal et al., 2012; Zhou et al., 2014; Shi et al., 2018). Methyl jasmonate can suppress the infection of *P. digitatum* on grapefruit by enhancing natural resistance (Droby et al., 1999).

Previously, several researchers have reported the potential of SA and MJ for the induction of defense mechanisms to protect treated fruit and vegetables from plant-pathogenic fungi (Cao et al., 2006; Iqbal et al., 2012; Zhou et al., 2014; Ali et al., 2022). Despite having tremendous antifungal potential for suppressing the onset of blue and green mold, these treatments are not systematically studied for the management of Penicillium rot of Citrus. There are only a few reports indicating the defense-inducing activity of resistance inducers against fungal diseases during post-harvest management.

The screening of antifungal botanical extracts has been widely conducted to develop plant based antifungal formulations that can be utilized to control post-harvest diseases of Citrus (Soylu et al., 2005; Talibi et al., 2012; Sukorini et al., 2013; Moosa et al., 2021). The use of plant extracts is safe, biodegradable and economical strategy for the control of plant diseases (Mamun and Ahmed, 2011). Management through plant extracts is a promising alternative control method due to its strong antimicrobial activity, non-phytotoxicity, and biodegradability (Tripathi and Dubey, 2004). Natural pesticides based on plant-dried spices and essential oils are gaining huge interest due to their safe nature. Spices-based plant extracts are generally non-phytotoxic and are potentially antimicrobial against several post-harvest pathogens (Isman, 2000; Bajpai et al., 2008). The antimicrobial properties of spices and their essential oils are widely recognized and they have been used to control several post-harvest diseases (Sukorini et al., 2013; Moosa et al., 2021). They contain a complex mixture of antimicrobial compounds such as tannins, coumarins, terpenoids, alkaloids, quinones, phenols, and flavonoids (Cowan, 1999; Ahmad et al., 2020). Moreover, these plant extracts are safe to human consumption and they are reported to promote human health (Veiga et al., 2020). However, the actual use of plant extracts and essential oils in post-harvest disease management is still limited.

The application of different control strategies in combination can provide more effective management of post-harvest diseases compared to stand-alone applications (Zhou et al., 2014; Moosa et al., 2021; Ren et al., 2021). Previously, Sukorini et al. (2013) reported that the combined application of yeast and medicinal plants suppressed the development of *P. digitatum* on tangerine fruit. The combination of *Bacillus amyloliquefaciens* and tea saponin (50 µg/mL) gave promising results to suppress the development of green mold, blue mold, and sour rot (Hao et al., 2011). In another instance, the growth of *P. digitatum* on mandarins (Guo et al., 2014) was suppressed through the preventive application of *Cryptococcus laurentii* and methyl jasmonate.

Keeping in view the suppressive potential of SA and plant extracts the study was designed; a) to reveal the efficacy of salicylic acid and plant extracts alone and in combination for suppressing the onset of blue and green mold of sweet oranges, and b) to explore defense enzymes activity in treated and untreated fruit.

## 2 Materials and methods

### 2.1 Fungal cultures

*Citrus sinensis* L. Osbeck cv. *“Succari”* fruit exhibiting typical symptoms of green and blue mold were picked from the local fruit market of Faisalabad, Pakistan, for the isolation of pathogens. The isolated fungal cultures *P. digitatum* Accession No. MH885505 and *P. italicum* (Accession No. MK08596) were preserved in 60% glycerol solution and stored at -80 °C.

### 2.2 Fruit

Disease-free, mature, and healthy sweet oranges were harvested from the Citrus Germplasm Unit, University of Agriculture Faisalabad, Pakistan. The fruit were surface sterilized with 1.0% NaOCl followed by rinsing with sterilized water. The surface-sterilized fruit were used for experimentation.

### 2.3 Preparation of plant extracts

Methanolic extracts of the following spices; *Cinnamomum verum* (bark), *Elletaria cardamomum* (dried fruit), *Cymbopogon citratus* (stem), *Curcuma longa* (rhizome), and *Syzgium aromaticum* (dried flower bud) were prepared. The plant parts were oven-dried for 24 h at 60 °C. Later, the dried plants were converted to powder and 300 g of powder was mixed in 4 mL of 98% methanol and incubated for 3 d in a rotary shaker. Post incubation, the suspension was passed through filter paper and the crude extract was taken. Then, the distillation of crude extract was carried out in rotavapor at 40 °C. The crude extract was dissolved in CH_2_Cl_2_ for 30 min at 1:3. After drying the extract was dissolved in 10% methanol and stored at 20 °C (Sukorini et al., 2013).

### 2.4 Antifungal assay

The methanolic plant extracts were tested for their antifungal activity against green and blue mold using the poison food method. Plant extracts were added to the PDA medium at 5, 10, and 15 g/L concentrations. A 5mm fungal culture block was excised from 1-week-old fungal culture and placed on the PDA medium amended with plant extracts. Post inoculation the Petri plates were incubated for 7 d at 25 ± 1 °C. Lactophenol blue solution was used to observe the colony growth of test fungi (Woo et al., 2010). Control treatment contained methanol only. The experimental procedure was repeated twice under the same conditions. All treatments consisted of 10 replicates. The growth inhibition of the test fungi was estimated by following the calculation procedure of Skidmore and Dickinson (1976). Cinnamon gave the best inhibition in an *in vitro* antifungal assay and was used for further study.

### 2.5 *In Planta* assay

On citrus fruit, SA and methanolic cinnamon extract were tested separately and together for their suppressive effects on the growth of pathogens. After washing with sterilized water, fresh fruit was surface sterilized with NaOCl 1.0% (v/v) and rinsed again with distilled water. The concentration of SA was 8mM which was chosen based on the findings of our previous research (Moosa et al., 2019). Later, 3 × 3mm wounds were artificially created in the peel of the fruit. Methanolic *C. verum* extract was prepared as described above in section 2.3. The fungal inoculum was prepared, and the spore density was adjusted to 10^5^ spores/mL using a hemocytometer. The treatment combinations were as follows; 1) CV = fruit dipping in sterilized water for 10 min + wound deposition of *C. verum* extract (10 μL) + wound deposition of sterilized water (10 μL), 2) SA = fruit dipping in SA solution for 10 min + wound deposition of sterilized water (10 μL), 3) GM= Inoculation of fruit with *P. digitatum* inoculum (10 μL), BM= Inoculation of fruit with *P. italicum* inoculum (10 μL), 4) HC= Healthy control fruit treated with wound deposition of sterilized water (10 μL), 5) SA + GM= fruit dipping in SA solution for 10 min + inoculation with *P. digitatum* inoculum (10 μL), 6) SA + BM= fruit dipping in SA solution for 10 min + inoculation with *P. italicum* inoculum (10 μL), 7) SA+ CV+ GM= fruit dipping in SA solution for 10 min + wound deposition of *C. verum* extract (10 μL) + inoculation with *P. digitatum* inoculum (10 μL), 8) SA+CV+BM = fruit dipping in SA solution for 10 min + wound deposition of *C. verum* extract (10 μL) + inoculation with *P. italicum* inoculum (10 μL). Post-treatment the fruit were kept in autoclaved plastic boxes. Then the fruit were kept in a 90% RH chamber at 25 ± 1 °C for 7 d. All treatments consisted of 15 replicates (1 fruit per replicate). Seven days post-incubation disease severity and disease incidence were measured. Disease incidence was recorded following the formula of Sukorini et al. (2013).


$$\text{Disease incidence }\left(\%\right)=\frac{\text{Number of decayed fruit}}{\text{Total number of examined fruit }}\times 100$$

Disease severity was assessed by measuring the lesion diameter on the fruit with a vernier caliper (Masood et al., 2010).


$$\text{Disease severity}=\;\frac{\text{Lesion diameter on fruit}}{\text{Total diameter of the fruit}}\times 100$$

### 2.6 Transcriptional analysis of defense enzymes

The transcriptional regulation of POD, PAL, and PPO genes was studied in sweet oranges. Total RNA from 3 g citrus fruit peel was extracted (Ballester et al., 2006) and added to RNase-free water. The concentration and purity of extracted RNA were tested on a NanoDrop1000 (Thermo Scientific, Wilmington, DA, USA). The first-strand cDNA was prepared by using the *Evo M-MLV* kit (Accurate Biology, Hunan, China) as per the manufacturer’s guidelines. A SYBR Green qPCR kit (Accurate Biology, Hunan, China) was used to evaluate the transcriptional regulation of defense enzymes. The gene expression was assessed in all treatments three days after incubation using QuantStudio RT-thermocycler (Thermo Fisher Scientific, CA, USA). The details and sequences of primers used in this project are listed in **Table 1**. For primer designing the sequences of respective defense enzyme genes were taken from the NCBI database available at (https://www.ncbi.nlm.nih.gov). The housekeeping *β-tubulin* gene was used to normalize the expression. The relative expression of all tested genes was calculated by using the 2-ΔΔct method (Livak and Schmittgen, 2001).

**Table 1:** List of primers and their sequences used in this study.

Code	Sequence (5^׳^–3^׳^)	Target gene	Size (bp)
*PAL-F*	CTTTGCCAGGCTATTGATTTGAGA	Phenylalanine ammonia-lyase	122
*PAL-R*	GATGGATGAAGCTCTCCACTAGC		
*POD-R*	ACAAGCAACTCTCTGCCCCC	Peroxidase	108
*POD-F*	AGGCGTTGGAGGTGATGAGG		
*PPO-F*	TACCGTGCCGGTGATGATCC	Polyphenol oxidase	143
*PPO-R*	ATAGGGTCTCTCCCAGCGGA		
*Tubulin-F*	TCCGCACTCTCAAACTCAGC	β-Tubulin	195
*Tubulin-R*	CACGGGATGTCAAGGGAGCA		

### 2.7 Enzyme activities assessment

The activity of PAL, POD, and PPO was measured in the fruit peel extract post-incubation for 3 d. The fruit peel was macerated in 0.2 M potassium phosphate buffer of pH 6.8. Then the obtained suspension was filtered through a sintered glass funnel followed by centrifugation at 12298 g for 10 min at 4 °C. The collected supernatant was used to measure the enzyme activity and total protein content (Coseteng and Lee, 1987).

To quantify the activity of POD guaiacol was used as a substrate. The reaction mixture for this assay was prepared by mixing 5 mM hydrogen peroxide, 5 mM guaiacol, and 0.2 M potassium phosphate buffer of pH 6.8. Subsequently, 800 μL of the reaction mixture and 200 μL of fruit peel extract were dissolved and the reaction was carried out. The absorbance was measured on a spectrophotometer at 470 nm by loading 100 μL of each sample in an Elisa microplate reader (Siegel and Galston, 1967). The total protein content of the samples was determined by using the protocol of Bradford (Bradford, 1976).

To determine PPO activity catechol was used as a substrate. To prepare the reaction mixture 50 mM catechol (500 μL), chilled acetone (500 μL), 0.2 M potassium phosphate buffer at pH 6.8 (2.5 mL), and fruit peel extract (200 μL). In the blank sample, only 1 mL of catechol was used. The absorbance was measured at 420 nm by loading the reaction mixture from each sample (100 µL) loaded in an Elisa microplate reader plate. The activity of PPO in each sample was expressed in min^-1^ kg^-1^ fresh weight unit. The enzyme activity was expressed as the total amount of enzyme that resulted in a change of 0.001 O.D. per min. in the absorbance.

The activity of PAL enzyme was measured by using acetone powder (Ballester et al., 2006). Citrus fruit peel 5 g was macerated in chilled acetone (50 mL) and the resulting suspension was filtered through a Buchner funnel. The collected residue was rinsed with chilled acetone solution and left for drying at room temperature for some time. The reaction mixture was prepared by mixing acetone powder (0.05 g), 20 mM mercaptoethanol, and 100 mM sodium borate buffer of pH 8.0 (1.5 mL). The supernatant was purified by using salting out proteins with 60% ammonium sulfate and desalted by using SephadexTM G-50 medium columns. The purified extract was recovered in 100 mM sodium borate buffer of pH 8.0 (1 mL). The reaction mixture consisted of purified enzyme (0.3 mL) and 0.1 M l-phenylalanine (0.1 mL). The absorbance was measured on a spectrophotometer at 290 nm wavelength. The activity of PAL was measured and expressed as nmoles g^-1^ h^-1^ of cinnamic acid.

### 2.8 Post-treatment fruit quality analysis

Seven days post-treatment the postharvest quality of the fruit was assessed. Total soluble solids (TSS) (%), titratable acidity (TA) (%), weight loss (%), and ascorbic acid (g/kg) of the treated and untreated fruit were determined. TSS was recorded in the fruit juice by measuring the refractive index on a digital refractometer and the final values were expressed in percentage. TA was determined by titration with 0.1 M sodium hydroxide of pH 8.3 and expressed in percentage (Hernández et al., 2006). Ascorbic acid was determined using the protocol of Roe et al. (1948). To calculate the percent weight loss the weight of fruit was recorded before treatment (A) and after treatment (B). The total weight loss of the fruit was calculated using the formula (A/B)/A × 100.

### 2.9 Statistical analysis

The obtained data from all experiments was processed for statistical analysis using the statistical package Statistix (ver. 8.1). After analysis of variance (ANOVA), the statistical differences of the treatment means were calculated using the LSD test at *p* ≤ 0.05.

## 3 Results

### 3.1 Antifungal assay

**Figure 1:**
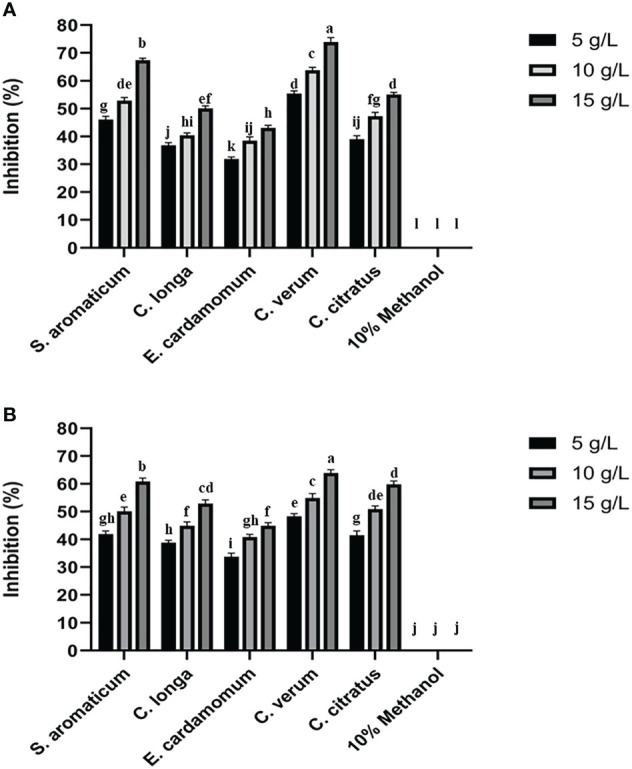
Effect of plant extracts on colony growth inhibition of P. digitatum **(A)** and P. italicum **(B)**. Error bars represent the standard error (± SE) of treatment means. Means with different alphabets over the columns indicate that the treatments are significantly different from each other.

The antifungal assay with 5 methanolic plant extracts was carried out *in vitro* at 5, 10, and 15 g/L concentrations against *P. italicum* and *P. digitatum*. Among all the tested plant extracts methanolic extracts of *C. verum* produced the highest mycelial growth inhibition of *P. italicum* and *P. digitatum* than the control treatment (**Figure 1**). The inhibition by *C. verum* was increased in a concentration-dependent manner with the highest inhibition at 15 g/L. Methanolic extracts of *S. aromaticum* produced the 2^nd^ highest inhibition of green and blue mold. All other tested plant extracts showed mycelial growth inhibition of green and blue mold.

### 3.2 *In Planta* experiment

The suppressive potential of SA and methanolic extract of *C. verum* against *P. digitatum* and *P. italicum* was evaluated on sweet oranges. SA and *C. verum* were tested stand-alone and in combinations. The treatment SA+CV caused the highest suppression of *P. italicum* and *P. digitatum* on sweet oranges compared to stand-alone treatments. SA+ *C. verum* showed the lowest disease incidence 26.7% of green and blue mold. Similarly, SA + *C. verum* exhibited the lowest disease severity 30.5% and 13.8% of *P. digitatum* and *P. italicum* respectively than control (**Table 2**).

**Table 2:** Effect of SA and *C. verum* on disease incidence and severity of green and blue mold.

Treatment	Green mold		Blue mold	
	Incidence (%)	Severity (%)	Incidence (%)	Severity (%)
SA	0.0 e	0.0 e	0.0 e	0.0 e
CV	0.0 e	0.0 e	0.0 e	0.0 e
Mold	100.0 a	55.8 a	100.0 a	43.9 a
SA + Mold	73.3 b	24.7 c	66.7 b	21.8 b
CV + Mold	60.0 c	30.5 b	33.3 c	13.8 c
SA + CV + Mold	26.7 d	15.9 d	26.7 d	6.8 d
HC	0.0 e	0.0 e	0.0 e	0.0 e

The values across the columns followed by the same alphabet are not statistically different from each other. Values are an average of 15 replicates analyzed by the LSD test at P ≤ 0.05. SA, Salicylic acid; CV, Cinnamomum verum; GM, Green mold; BM, Blue mold; HC, Healthy control.

### 3.3 Transcriptional analysis of defense enzymes

**Figure 2:**
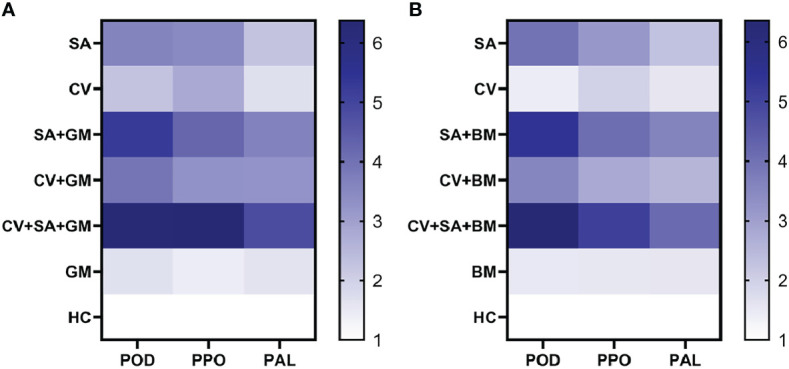
Heat map for the relative expression of PPO, POD, and PAL in sweet oranges. **(A)** Relative expression of PPO, POD, and PAL against green mold, **(B)** Relative expression of PPO, POD, and PAL against blue mold. PPO= Polyphenol oxidase, POD, Peroxidase; PAL,Phenyl alanine ammonia-lyase; SA, Salicylic acid; CV, Cinnamomum verum; GM, Green mold; BM, Blue mold; HC, Healthy control.

To unravel the role of defense enzymes *POD*, *PPO*, and *PAL*, the relative expression of respective genes was measured in a real-time PCR assay. The ability of SA and methanolic extract of *C. verum* was tested to bring about changes in the relative expression of *POD*, *PPO*, and *PAL* genes. The treatment SA+CV caused the highest increase in the expression of all three defense genes in fruit peel. The heat maps in **Figure 2** indicate the changes in the relative expression of all three genes based on a color chart. The expression of all genes was higher in fruit treated with SA + CV 3 d post-incubation than the control treatments (**Figure 2**). The expression of *POD* was 6.3-fold high compared to control in fruit treated with SA + *C. verum* against *P. digitatum* and *P. italicum*. *PPO* showed a 6.4-fold and 5.1-fold increase against *P. digitatum* and *P. italicum* respectively. Similarly, PAL expression was also 4.1 and 4.8-fold enhanced against *P. digitatum* and *P. italicum* respectively than the control. The most upregulated defense gene was POD in the case of all treatments. Stand-alone application of SA and CV also showed an increase in the relative expression of all tested defense genes.

### 3.4 Defense enzyme activities assessment

**Figure 3:**
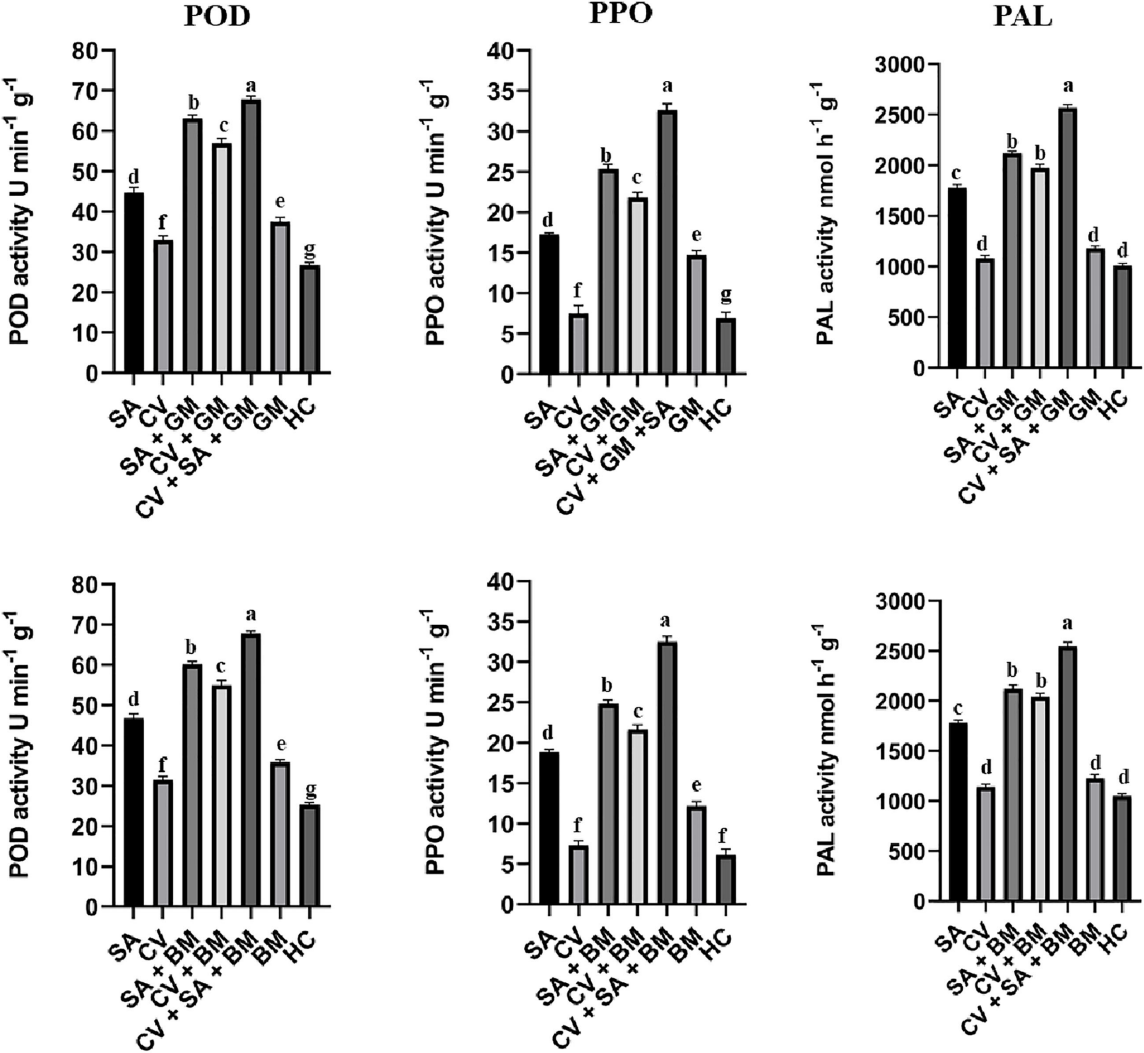
Activity of PPO, POD, and PAL in sweet oranges treated with combined and stand-alone applications of SA and C. verum. Error bars represent the standard error (± SE) of treatment means. Means with different alphabets over the columns indicate that the treatments are significantly different from each other. PPO, Polyphenol oxidase; POD, Peroxidase; PAL, Phenyl alanine ammonia-lyase; SA, Salicylic acid; CV, Cinnamomum verum; GM, Green mold, BM, Blue mold, HC, Healthy control.

The product of POD, PPO, and PAL was quantified by measuring absorbance on a spectrophotometer. The quantification assay revealed a similar pattern as observed in the relative expression of respective genes. The treatment SA + CV produced the highest amount of POD, PPO, and PAL in treated fruit peel at 3 d post-incubation against both molds than the control (**Figure 3**). The activity of POD, PPO, and PAL was also increased in fruit treated with the stand-alone application of SA and *C. verum*. Furthermore, PPO, POD, and PAL activity were also slightly higher in untreated fruit infected with *P. digitatum* and *P. italicum* than in the healthy control. The results have suggested the defense activating potential of SA and *C. verum* against green and blue mold. The uninoculated sweet oranges treated with stand-alone applications of SA and *C. verum* also exhibited a rise in POD, PPO, and PAL activity.

### 3.5 Post-treatment quality analysis of the fruit

The effect of treatments on the post-harvest quality of sweet oranges was assessed. Fruit treated with SA + CV showed the lowest change in weight loss, ascorbic acid, total soluble solids, and low titratable acidity compared to healthy and infected control fruit (**Table 3**). The quality of fruit was affected in sweet oranges inoculated with *P. digitatum* and *P. italicum* only. Treatment with SA and *C. verum* alone and in combinations suppressed the disease along with the lowest impact on fruit quality.

**Table 3:** Post-harvest quality of sweet oranges in response to treatment with SA and cinnamon.

Treatment	Weight loss (%)	Ascorbic acid (g/100kg)	Titratable acidity (%)	Total soluble salts (%)
***P. digitatum***				
SA	0.00 e	13.80 e	0.82 c	7.40 e
CV	0.00 e	13.70 e	0.86 a	7.20 e
SA+GM	2.72 b	16.26 b	0.29 e	11.00 c
CV+GM	2.14 c	15.80 c	0.28 e	12.60 b
CV+SA+GM	0.72 d	14.61 d	0.44 d	10.10 d
GM	0.00 e	18.13 a	0.20 f	13.10 a
HC	3.76 a	13.50 e	0.84 b	7.50 e
***P. italicum***				
SA	0.00 e	14.15 d	1.04 a	7.80 d
CV	0.00 e	13.98 d	1.04 a	7.65 d
SA+BM	2.42 b	16.67 b	0.32 c	12.20 b
CV+BM	2.10 c	16.00 bc	0.31 c	12.03 b
CV+SA+BM	0.87 d	15.00 cd	0.43 b	9.42 c
BM	3.67 a	20.00 a	0.23 d	13.20 a
HC	0.00 e	14.31 d	0.99 a	7.90 d

The values across the columns followed by the same alphabets are statistically not different from each other. Values are an average of 15 replicates analyzed by LSD test at P ≤ 0.05. SA, Salicylic acid; CV, Cinnamomum verum; GM, Green mold; BM, Blue mold; HC, Healthy control.

## 4 Discussion

This study presents the promising suppressive potential of salicylic acid in combination with cinnamon against green and blue mold of sweet orange. Chemical, physical, and biological agents have the potential to induce local and systemic defense responses in plants against several pathogens. The use of resistance inducers has achieved great research attraction in recent years (Quaglia et al., 2011; Iqbal et al., 2012; Romanazzi et al., 2016). The induction of resistance does not provide complete control of the disease, however, it reduces the disease intensity by triggering the defense responses (Walters et al., 2005). The suppressive effect of chemical resistance inducers can be improved by using them in combination with other management strategies. The application of salicylic acid and cinnamon alone and in combinations reduced the development of green and blue mold on sweet oranges.

In a previous report (Moosa et al., 2019) we reported the inhibitory effect of SA on the green and blue mold of mandarin. In continuation with the findings of that report, we evaluated the effect of SA in combination with *C. verum* extract against the green and blue mold of mandarin cv. ‘Kinnow’ (Moosa et al., 2021). In this study, we have tested the same combinations of SA and cinnamon against green and blue mold infection on sweet oranges cv. ‘Succari’. We hypothesized that the integration of SA and cinnamon can enhance the suppressive potential of SA against *P. digitatum* and *P. italicum*.

The *in vitro* screening of five methanolic plant extracts revealed that *C. verum* was the most inhibitory against green and blue mold. The antifungal effect of cinnamon has been reported against postharvest rots in several previous studies (Wang et al., 2019; Aguilar‐Veloz et al., 2020; Tahmasebi et al., 2020). Botanical extracts have exhibited a remarkable suppressive potential against post-harvest fungal rots (Obagwu and Korsten, 2003; Askarne et al., 2013; Shao et al., 2013; Sukorini et al., 2013). Cinnamon contains cinnamaldehyde and eugenol that contribute to its promising antifungal activity (Singh et al., 1995; Simić et al., 2004; Jantan et al., 2008). According to several previous reports, cinnamaldehyde is a potential compound for the development of antifungal formulations against molds which directly controls chitin synthesis and β-(1,3)-glucan (Bang et al., 2000). Therefore, we have concluded that cinnamaldehyde is the principal antifungal compound in cinnamon.

In an *In Planta* assay SA and cinnamon alone and in combination reduced the disease development of green and blue mold citrus fruit. The suppressive effect of SA and cinnamon was considerably enhanced when they were applied in combination. The synergistic antimicrobial action of SA in combination with other treatments such as chitosan (He et al., 2011; Shi et al., 2018), *Pichia membranaefaciens* (Zhou et al., 2014), and yeast (Zhang et al., 2008) has been reported previously. Similarly, in our previous study, we reported that the antifungal potential of SA and *C. verum* against green and blue mold on mandarin fruit was remarkably enhanced when they were applied in combination (Moosa et al., 2021). Previously, Sukorini et al. (2013) reported that a combination of clove extract and yeast caused the highest reduction in lesion diameter of green and blue mold on citrus fruit. The inhibitory effect of plant extracts is mainly attributed to the presence of antifungal compounds such as phenolics, flavonoids, terpenoids, tannins, and alkaloids in plants (Cowan, 1999) while the resistance inducers elicit and modulate the activity of defense enzymes in plants which ultimately contribute to enhanced disease suppression (Iqbal et al., 2012; Zhou et al., 2014). Moreover, it was observed that the combination of SA and *C. verum* in our study showed no effect on the post-harvest quality parameters i.e., weight loss, ascorbic acid, total titratable acidity, and total soluble solids of sweet oranges. In agreement with our findings, (Sukorini et al., 2013) also stated that post-harvest treatment of citrus fruit with a combination of clove and yeast against green and blue mold did not affect the fruit quality. In another study, a combination of SA and chitosan suppressed green mold and did not impair fruit quality (Shi et al., 2018). We have concluded that the integration of SA and cinnamon extract can provide better control of green and blue mold rot of citrus without affecting the post-harvest quality of citrus fruit.

Furthermore, to unravel the defense mechanism underlying the suppression of green and blue mold of sweet oranges the transcriptional analysis of major defense enzymes *PPO*, *POD*, and *PAL* was conducted. Transcriptional analysis revealed that the relative expression of *PPO*, *POD*, and *PAL* genes was upregulated in sweet oranges treated with a combination of SA and *C. verum* compared to healthy and infected controls. In support of our work, Chen et al. (2019) reported that citrus fruit treated with clove essential oil showed a reduction in the lesion diameter of blue mold and upregulated the *PPO*, *POD*, and *PAL* genes. In this study, SA also caused an upregulation in the relative expression of *PPO*, *POD*, and *PAL*. In a previous report, SA caused an increase in phenylalanine ammonia-lyase (PAL) and β-1,3-glucanase compared to control fruit against anthracnose of mango (Zeng et al., 2006). In our study, a correlation between *PPO*, *POD*, and *PAL* expression, and enhanced resistance against green and blue mold of citrus has been observed. We concluded that the increased expression of *PPO*, *PAL*, and *POD* can be possibly involved in the suppression of green and blue mold.

To validate the expression analysis of *PPO*, *POD*, and *PAL* genes the respective gene products were quantified using a spectrophotometer. Similar to the results obtained in transcriptional analysis the activity of PPO, POD, and PAL was highest in fruit treated with a SA + CV. Our findings suggest that SA and *C. verum* increased the activity of PPO, POD, and PAL to suppress green and blue mold infection on sweet oranges. The induction of resistance in harvest fruit is an effective method to enhance the suppression of post-harvest rots caused by fungal pathogens (Cao et al., 2006). PPO is a vital enzyme involved in plant defense responses that plays an integral role in the lignification of plant cells and it converts phenols to toxic quinones which contribute to its high toxicity against several invading pathogens (Wallace and Fry, 1999; Mohammadi and Kazemi, 2002). Our results showed a positive correlation between the enhanced activity of PPO and the suppression of green and blue mold suggesting its role in plant defense. POD is another important defense enzyme that catalyzes the final step in the formation of lignin and converts the phenolic compounds to highly toxic quinones. In a previous report, an increase in the activity of PPO and POD was observed in citrus fruit treated with a combined application of methyl jasmonate and yeast (Guo et al., 2014). PAL is involved in the regulation of the phenolic pathway and catalyzes the conversion of phenylalanine to trans-cinnamate. Moreover, PAL is a precursor of scoparone and scopoletin which are reported to be antifungal compounds against several fungal pathogens (Droby et al., 2002). The enhanced resistance in sweet oranges against green and blue mold might be due to the increased activity of PAL. Several previous reports have stated that an increase in the activity of PPO, PAL, and POD conferred enhanced resistance against fungal pathogens (Qin et al., 2003; Shao et al., 2013; Shi et al., 2018; Moosa et al., 2021). Therefore, the present study implies that PAL, PPO, and POD defense enzymes are possibly connected with enhanced defense of sweet oranges against green and blue mold diseases.

## 5 Conclusion

Conclusively, the present study discloses the promising suppressive potential of SA and cinnamon by upregulating the expression and activity of defense enzymes against green and blue mold of sweet oranges. The integration of SA and cinnamon showed a remarkable antifungal activity comparable to synthetic chemicals to suppress the development of green and blue mold. The suppressive potential of SA and *C. verum* and the underlying mechanisms must be further explored to validate the possible inclusion of these treatments in the integrated disease management program for Penicillium rot of citrus.

## Data availability statement

The raw data supporting the conclusions of this article will be made available by the authors, without undue reservation.

## Author contributions

AM conceived and performed the experiment. AM and FZ wrote the first draft. All authors review and revised the manuscript and approved the final version of the manuscript.

## Conflict of interest

The authors declare that the research was conducted in the absence of any commercial or financial relationships that could be construed as a potential conflict of interest.

## Publisher’s note

All claims expressed in this article are solely those of the authors and do not necessarily represent those of their affiliated organizations, or those of the publisher, the editors and the reviewers. Any product that may be evaluated in this article, or claim that may be made by its manufacturer, is not guaranteed or endorsed by the publisher.
